# Diagnosis of coronary artery disease using targeted post-mortem computed tomography coronary angiography: a case report

**DOI:** 10.1080/20961790.2017.1328795

**Published:** 2017-06-05

**Authors:** Lei Wan, Yu Shao, Donghua Zou, Ping Huang, Zhengdong Li, Maowen Wang, Yijiu Chen

**Affiliations:** Shanghai Key Laboratory of Forensic Science, Shanghai Forensic Service Platform, Department of Forensic Pathology, Institute of Forensic Science, Ministry of Justice, Shanghai, China

**Keywords:** Forensic science, forensic pathology, post-mortem forensic imaging, PMCT, PMCTA, targeted coronary angiography, coronary atherosclerosis

## Abstract

Targeted post-mortem computed tomography (PMCT) combined with coronary angiography has the potential to play a significant role in the investigation of sudden cardiac death. The authors utilized a targeted PMCT coronary angiography in a case involving a 53-year-old man who died from acute myocardial ischemia and cardiac decompensation which may result from coronary artery disease (CAD). The victim collapsed suddenly at work and died soon after arrival to hospital. The body was examined using PMCT and targeted PMCT coronary angiography. The left anterior descending coronary artery exhibited 75%–100% stenosis in the middle segment; however, the distal segment could not be clearly visualized. In addition, the left circumflex and right coronary artery exhibited calcification, atherosclerosis and an area of 50% stenosis. Signs of cardiogenic pulmonary oedema were also identified. The imaging results suggested that this individual had coronary atherosclerosis and probably died from CAD. The autopsy and histological examination revealed acute myocardial ischemia and myocardial scarring, confirming the cause of death while excluding other probabilities. In summary, targeted post-mortem computed tomography angiography (PMCTA) can visualize the arteries and estimate the degree of principal pathological changes. This method is a simple, reliable and sensitive technique for identifying the presence of coronary atherosclerosis. It is a valuable post-mortem forensic imaging method and should be recommended in the investigation of suspicious cardiac deaths.

## Introduction

The number of deaths from cardiovascular disease has rapidly increased worldwide and coronary artery disease (CAD) accounts for approximately 80% of sudden cardiac deaths [1]. For centuries, autopsy has been the most commonly used tool for evaluating sudden cardiac death. Because the conventional autopsy may often be denied by family members or not tolerated by certain religions, there is an urgent need to develop an alternatively non-invasive/minimally invasive technique.

In recent years, medical imaging has provided a safe, convenient means for diagnosis of diseases in forensic examination. Recently, targeted post-mortem computed tomography (PMCT) combined with coronary angiography, used in the United Kingdom, demonstrated high sensitivity and specificity [2–6]. Two popular types of targeted post-mortem computed tomography angiography (PMCTA) have been reported as the Oxford method [2] and the Leicester method [3]. Here, we report a case in which a sudden death due to CAD was confirmed by the combination of PMCT, coronary angiography, autopsy and histology, supporting the value of PMCT and targeted PMCTA in the diagnosis of CAD.

## Case report

A 53-year-old man collapsed suddenly at work and was taken to hospital, where he presented respiratory and cardiac arrest. Adrenalin and other rescue medications have been used. No vital sign was revealed through the resuscitation and death was declared soon after. A forensic examination was performed two weeks after death. Since forensic autopsy of the suspected deaths due to illness are not forced by law in China. And such autopsy must be granted by the relatives, which may lead to the very long time interval between the autopsy and death. The victim was 175 cm in height and had a normal body shape. Permission for PMCT, PMCTA and autopsy was granted by the victim's relatives.

## PMCT and PMCTA examination

PMCT was performed after the external examination. The whole-body PMCT scan was performed using a 40-slice multislice CT system (Definition AS; Siemens Medical Solutions, Munich, Germany). Raw data were acquired using the following settings: voltage 120 kV; current 240 mAs and collimation 6.0 mm × 1.0 mm. Image reconstruction was achieved at slice thicknesses of 5.0 mm and 0.625 mm, each with an increment of half the slice thickness (soft tissue and bone-weighted reconstruction kernel). Image review and three-dimensional reconstructions were performed using a CT workstation (Syngo Imaging XS; Siemens Medical Solutions).

For targeted PMCTA, the angiography protocol used was a combination of the above-mentioned Leicester method and Oxford method. According to the operating procedures of the Oxford method [2], cut down into the left common carotid artery was made and a three-way urinary catheter with a 30 mL balloon was inserted. Before injecting contrast medium, the position of the catheter tip was ascertained using CT and repositioned if necessary to reach a position exactly above the aortic valve. The balloon was fully inflated. Then 150 mL of contrast medium (diatrizoate meglumine: 0.9% normal saline [10:1] used in the Leicester method [3]) was injected manually at a rate of 50 mL/8 s. Scanning was performed directly after administering the contrast medium.

## Autopsy and other analysis

A traditional autopsy was performed about one hour after the PMCTA examination. Peripheral blood taken by femoral puncture was sent for toxicological analyses before the contrast agent injection in the PMCTA procedure. During autopsy, external and internal examinations of the body were performed. Histology samples of most of the organs within the cranial, thoracic and abdominal cavity were taken and processed in H&E staining.

## Radiological findings

During PMCT, the proximal segment of the left anterior descending coronary artery (LAD) exhibited severe calcification; the left circumflex coronary artery (LCX) and right coronary artery (RCA) exhibited diffuse calcification. Additionally, a pulmonary ground-glass opacity and small amount of fluid in the subglottic trachea and main bronchi were identified. No other abnormalities were found.

**Figure 1.**
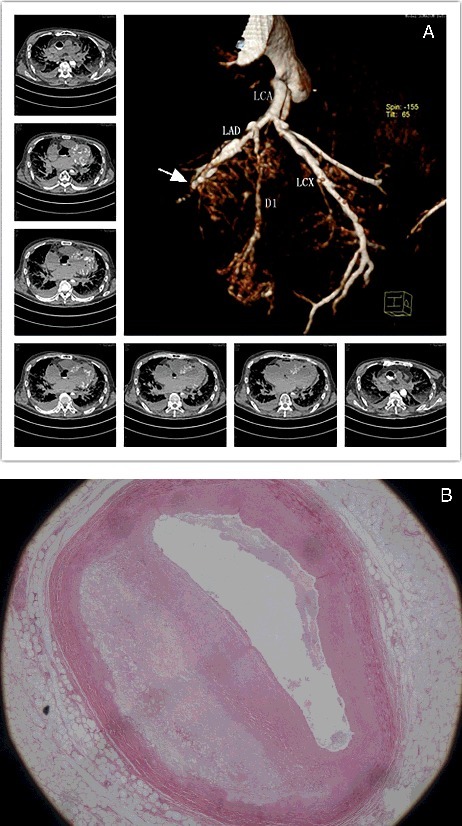
(A) Volume-rendered contrast-enhanced image revealing contrast medium filling the aortic root and the LAD. The LAD had 75%–100% stenosis in the middle segment; however, the distal segment could not be clearly visualized (arrow). (B) Histological examination confirming 75%–100% stenosis in the middle segment of the LAD. (H&E ×20).

**Figure 2.**
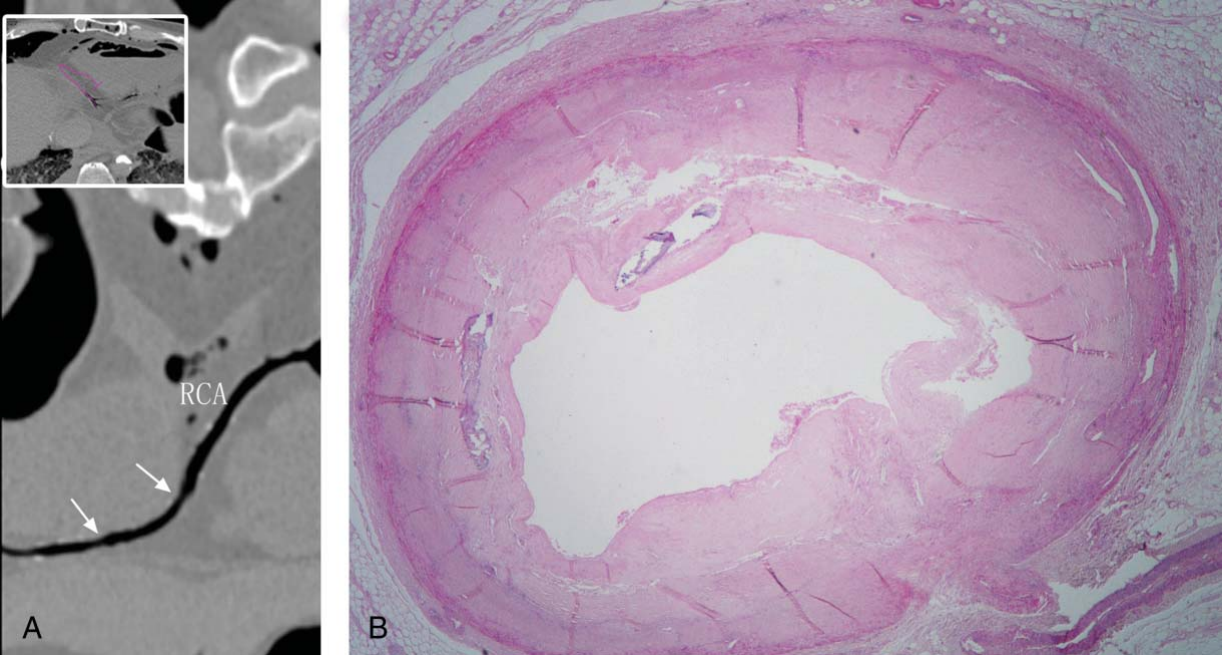
(A) Curved multiplanar reconstruction image revealing calcification and atherosclerosis of RCA, with several localized area exhibiting 50% stenosis (arrows). RCA is marked and appears in black for it is filled with gas. (B) Histological examination confirming 50% stenosis in the RCA. (H&E ×20).

Although the RCA was filled with air during targeted PMCTA, good visualization of the lumen was still possible. The LAD exhibited 75%–100% stenosis in the middle segment; however, the distal segment could not be clearly visualized (Figure 1(A)). The LCX and RCA exhibited calcification, atherosclerosis, with a localized area of the RCA exhibiting 50% stenosis (Figure 2(A)).

## Autopsy, histological findings and toxicological analysis

**Figure 3.**
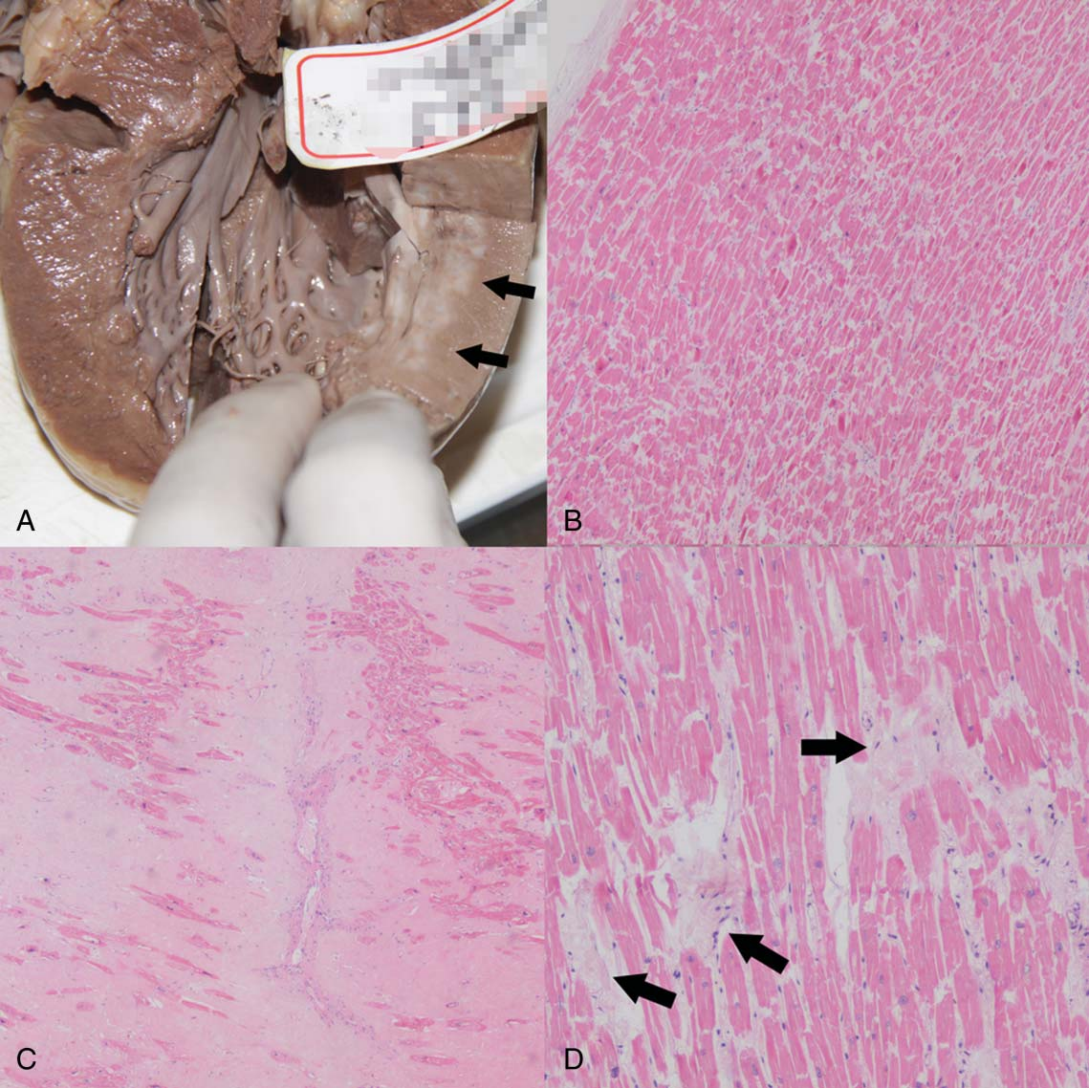
**(A**) Autopsy revealed myocardial fibrosis in the posterior wall of the left ventricle (arrows). Histological examination of samples in H&E staining revealed (B) enhanced eosinophilic staining and fractures of cardiomyocytes (×40), (C) myocardial scarring (×20) and (D) multiple dissolution of cardiomyocytes (arrows) (×100).

During autopsy, only minor abrasions were found in the extremities. The weight of the heart was 628 g, with left ventricular hypertrophy and cardiac dilation. Partial myocardial fibrosis was found, especially in the posterior wall of the left ventricle (Figure 3(A)). Coronary atherosclerosis was found with stenosis in LAD, RCA and LCX. No sign of fresh myocardial infarction was found in macroscopy. The left lung was 961 g and the right lung was 1186 g in weight. Pulmonary congestion and edema were revealed. No other abnormalities were found.

Histological examination revealed acute myocardial ischemic changes including enhanced eosinophilic staining and considerable fractures of cardiomyocytes, dissolution of cardiomyocytes and myocardial scarring (Figure 3(B–D)). Typical atherosclerotic changes were found, and the degree of calcification and stenosis were consistent with the PMCT and PMCTA results. The LAD exhibited 75%–100% stenosis in the middle segment (Figure 1(B)), while the LCX and RCA (Figure 2(B)) had 50% stenosis. Pulmonary oedema and blood congestion of multiple organs were revealed, together with intimal thickening of arteries in spleen and kidneys.

No other abnormalities were found and toxicological analysis result was negative.

According to the PMCT, PMCTA, autopsy and histological findings while combined with the sudden death process of the victim and ruling out other probabilities, the cause of death was determined to be acute myocardial ischemia and cardiac decompensation resulting from CAD.

## Discussion

The most common cause of cardiovascular disease-related death is coronary atherosclerosis. Sudden death is, in fact, the initial symptom in approximately 25% of individuals dying from coronary atherosclerosis [1]. Arterial stenosis caused by coronary atherosclerosis is a basic pathological change observed in CAD, and calcification can be evaluated using radiological approaches [7].

Currently, there have been significant improvements in the quality of multiplanar reconstruction in CT imaging. Being an accepted, non-invasive radiological imaging technique, PMCT has been considered as an alternative to autopsy in certain scenarios. While PMCT is generally considered to be a good complement for conventional autopsy, it was thought to have limited application in cardiovascular pathology [8–9]. To date, however, it has not often been used separately from autopsy for determining the cause of death in sudden cardiac deaths [10].

Researches concerning the use of PMCTA in the investigation of sudden cardiac deaths have been reported [6,8,11–13]. There are several principal forms of PMCTA: a whole-body infusion angiography technique reported from Switzerland [12,14–15]; cardiopulmonary resuscitation to establish circulation in Japan [16–17] and single-organ approaches, such as targeted coronary angiography, used in the United Kingdom [2–3,6]. These PMCTA techniques are distinguished according to delivery and type of contrast agents and their targets. In addition, numerous studies have focused on performing PMCTA on the heart or brain via catheters, which appears to have some advantages [2,3,5,18].

The above-mentioned Leicester method and Oxford method differ in their approach. The Oxford method uses only positive contrast media and makes efforts to avoid air within the vessels. The Leicester method deliberately uses both air and contrast media. Another difference is the use of pump injector system to image the coronary vessels dynamically during contrast injection by the Leicester team [6]. The targeted PMCTA method in the present case was a mixture of the two popular and mature methods. Operating procedures of the Oxford method were combined with contrast medium and dilution used in the Leicester method, forming a simple, reliable and sensitive technique for the diagnosis of CAD.

In the present case, according to the reported circumstances of death, we suspected a sudden cardiac death due to CAD. Therefore, PMCT and targeted coronary angiography were performed. PMCTA revealed severe coronary atherosclerosis and stenosis of the LAD, supporting our suspicion about the cause of death. Targeted coronary angiography only allows investigating the coronary arteries and estimating the degree of pathological changes, making the diagnosis of CAD. Deterministic evidences like myocardial ischemia or infraction in macro- and micro-scopic investigations must be found to confirm the cardiac death, while excluding other causes of death. During autopsy and histological examination, more attention was devoted to the heart, and the calcification and stenosis of the coronary arteries were highly consistent with the PMCT and PMCTA results. In addition, the pulmonary ground-glass opacity and a small amount of fluid in the subglottic trachea and main bronchi suggested cardiogenic pulmonary oedema, which is common in the case of sudden cardiac death [19]. According to the combination of PMCT, PMCTA, autopsy, and histological findings while ruling out other probabilities, the cause of death was confirmed.

The multiphase PMCTA (MPMCTA) from Switzerland is currently the best evaluated post-mortem angiography method to precisely localize lesions even from very small vessels. Vascular occlusions in cases of myocardium infarction can be detected with high diagnostic confidence. Besides the analysis of coronary arteries, it has also proved useful for the detection of myocardium infarctions [20]. Pathologic enhancement of the myocardium (mean Hounsfield Units ≥ 95) is thought to be an indirect sign of a myocardial lesion if the technique of MPMCTA is applied and the certain contrast medium is used [12]. Also in targeted coronary PMCTA, an associated perfusion deficit in myocardium associated with the concerned vessel is helpful but not specific to infarction as perfusion deficits may be seen in normal myocardiu [6]. Unfortunately, in the present case no such sign of myocardium infarction was revealed.

There are potential pitfalls associated with using *in situ* PMCTA, including introduction of air into vessels, resulting in incomplete filling with contrast medium. Efforts have been made to avoid such filling defects [6,12,21]. In the present case, the contrast medium was injected into the left coronary artery, while the RCA was filled with air. The presence of air acts as a negative contrast agent and does not necessarily affect the diagnosis. In fact an air angiogram still enabled visualization of the arterial lumen.

In summary, targeted PMCTA can be used to visualize the arteries and estimate the degree of principal pathological changes. This method is simple, reliable and sensitive for identifying CAD. The targeted PMCTA approach in this study demonstrated that radiological findings correlate well with autopsy and histological findings with regard to the coronary arteries. This method can be a valuable adjunct to the post-mortem radiological examination and is applicable to a routine diagnostic protocol.
